# Dynamic Time Warping for System-Level Fault Detection in IoT Devices: An Episode- and Layer-Based, Label-Free Approach

**DOI:** 10.3390/s26123920

**Published:** 2026-06-20

**Authors:** Ryan Aalund, Vincent P. Paglioni

**Affiliations:** Risk, Reliability, & Resiliency Characterization Lab, Department of Systems Engineering, Colorado State University, Fort Collins, CO 80523, USA; vincent.paglioni@colostate.edu

**Keywords:** IoT, fault detection and diagnosis, anomaly detection, dynamic time warping, time series, label-free learning

## Abstract

IoT devices operate as integrated systems spanning hardware, firmware/software layers, and communication layers. In operational settings, many faults and performance degradations are emergent: they arise from cross-layer interactions, workload changes, and telemetry artifacts, rather than a single physics-of-failure mechanism. These realities make traditional supervised fault classification difficult because labeled fault data are rarely available during deployment, and the fault surface is unknown and a priori. This paper presents a practitioner-oriented, label-free fault detection and diagnosis (FDD) pattern based on Dynamic Time Warping (DTW) for rapid implementation in production IoT telemetry. The method represents a device as a sequence of overlapping episodes and organizes telemetry into interpretable layers (hardware sensors, communication health proxies, and software/firmware-derived KPIs). A reference library of regular episodes is built from an assumed-healthy training window; new episodes are scored using constrained DTW distances against this library, while retaining per-layer and per-channel contributions for attribution. We show that production performance depends strongly on operational parameterization, including episode length, DTW constraints, robust threshold learning, and temporal validation. Within a verified-healthy evaluation window, the tuned configuration achieves an AUROC of 0.97 for the temporally structured faults DTW is suited to (bias, drift, and interaction faults, with spikes detected at an AUROC of 0.93), detecting 100% of injected faults, with a mean delay under 25 min. We further show that constant-value (stuck-at) and missing-data (dropout) faults fall outside DTW’s shape-matching scope (AUROC about 0.66) and are better served by complementary variance- and missingness-based detectors, a consequence of DTW’s shape-matching scope rather than a parameter choice. This work contributes a system-level methodological framework for deploying DTW as an IoT fault-detection-and-diagnosis capability: an episode-and-layer architecture aligned with hardware, communication, and software/firmware ownership; a label-free reference library requiring only assumed-healthy data; per-layer and per-channel attribution for cross-domain triage; and a reproducible operational tuning procedure. Together, these deliver a fast-to-deploy, scalable, and accurate first-line detector for label-scarce IoT systems.

## 1. Introduction

Internet of Things (IoT) devices and cyber-physical systems increasingly underpin industrial automation, building management, energy, transportation, and consumer infrastructure. These systems are complex, highly interdependent networks of components encompassing hardware, software/firmware, and communications capabilities. Accordingly, the detection and diagnosis of faults in the system is critical to preventing cascading failures and potentially significant consequences. Regardless of domain, IoT and cyber-physical systems generate multi-sensor telemetry streams that must be monitored continuously to detect faults, degradations, and security-relevant anomalies. Despite sustained progress in anomaly detection and fault diagnosis, operational IoT monitoring remains challenging because real systems seldom match textbook assumptions: telemetry is irregular, missing, or seasonally driven; device configurations evolve; and many critical failures are rare, novel, or poorly labeled in production data [1,2].

A significant gap between research and practice is the dominance of assumptions about supervised learning. In many industrial and IoT settings, comprehensive labeled fault datasets are unavailable, and the organization may not know in advance which faults will manifest or where they will appear (sensor, firmware, application logic, or network). Reviews of industrial fault diagnosis and predictive maintenance consistently highlight data scarcity, label limitations, and the need for methods that work with weak or no supervision [3,4]. Clearly, there are multifold challenges to efficient and effective fault detection and diagnosis (FDD) in IoT and cyber-physical systems. At a foundational level, there is a clear gap in understanding and approach. The assumed primacy of supervised learning as a necessary and sufficient component of FDD has limited researchers’ and practitioners’ ability to develop and implement novel approaches to FDD. This translates to a practical challenge: implementing FDD approaches with an inadequate data stream, despite the plethora of data points.

This paper addresses the typical practitioner’s need: deploying a functional fault-detection capability quickly, with minimal labeling, and with outputs that guide debugging across layers of the IoT or cyber-physical system. The IoT system is modeled as a system consisting of (i) hardware sensing and actuation, (ii) communication and data integrity, and (iii) software/firmware behavior as reflected in available telemetry. Many operational faults are emergent and cross-layer; they may not follow a single physics-of-failure model but instead arise from interactions, such as timing drift with packet loss or firmware duty-cycle changes interacting with sensor nonlinearity [5]. Intermittent and context-dependent faults further complicate diagnosis [6].

To achieve a functional, expedient, and operational FDD approach for IoT and cyber-physical systems, this paper employs Dynamic Time Warping (DTW) within an episodic, layer-based system-level architecture. DTW is widely used to measure similarity between time series with temporal misalignment and varying rates [7,8]. DTW can compare the shape of episodic behaviors without requiring aligned timestamps or labeled fault classes, making it attractive for early-stage IoT and generally label-constrained deployments. However, practical guidance is needed on deploying DTW at the system level, engineering cross-layer features, scoring and thresholding episodes, and extracting actionable attribution for diagnosis. This work offers the missing guidance through an integrated, system-level framework that transforms DTW into a deployable, label-free, and interpretable detector. It shows how the operational parameters can be set from deployment criteria, without labeled or evaluation data. The approach is demonstrated on publicly available IoT telemetry with synthetic fault injection and visual explanations.

Section 2 of this paper provides a brief background on IoT and cyber-physical systems and their challenges, and reviews DTW and related approaches. Section 3 provides more details on the problem space and the proposed solution architecture, which is fully specified in Section 4 as a DTW methodology and corresponding case study. Section 5 presents the results of the methodology as applied to the case study. Section 6 discusses the method’s practical value, limitations, and deployment guidance, and Section 7 concludes.

## 2. Background and Related Work

IoT and cyber-physical systems are increasingly vital to the global economy, underpinning operations across domains such as industrial automation, energy production and transmission, transportation, and consumer infrastructure. Beyond traditional embedded system architectures, IoT and cyber-physical systems integrate heterogeneous hardware, firmware, software, and communication components across dynamic and harsh operating environments. In these environments, these systems must function autonomously and with high reliability to avoid significant hazards, economic losses, and/or cascading failures across critical infrastructure [9]. Considering the potential consequences, it is imperative to understand the challenges to reliability in IoT and cyber-physical systems, particularly those related to fault detection and diagnosis, to help mitigate cascading failures in tightly coupled systems.

### 2.1. IoT Systems and Challenges

**Figure 1:**
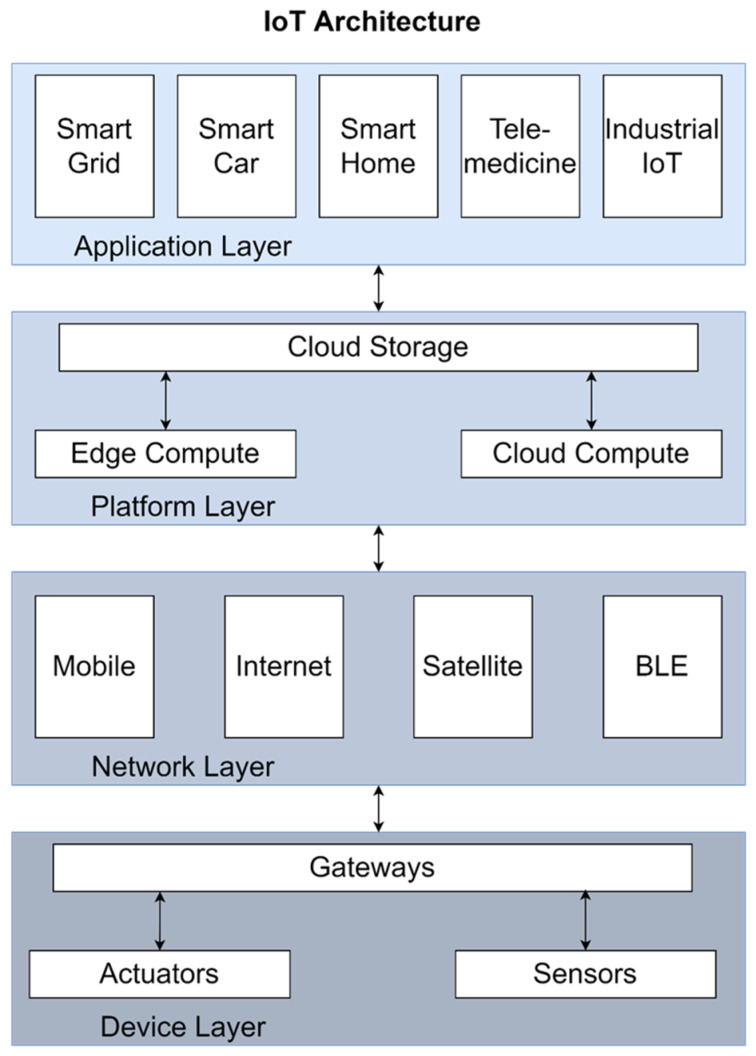
Layered IoT system architecture. Arrows represent interactions between layers and interlayer components. Adapted from Lee & Seshia [9].

A common heuristic for conceptualizing IoT and cyber-physical systems is a layered model [10], as in Figure 1. This perspective highlights the multi-domain, multi-level nature of IoT systems, which leverage hardware, software, and firmware operating in concert and communicating across multiple levels (e.g., application, platform, network, and device) to accomplish some system function. IoT systems are, thus, highly complex and tightly coupled, with continuous interactions between layers and components. This tight coupling means that the system can process and respond to the rapid dynamics that often characterize the operating environment, but also allows for failure cascades [11].

IoT systems present significant challenges for establishing and maintaining system reliability. Fundamentally, the embedded, complex dynamics of most IoT systems make it difficult to identify faults and trace their root causes, which may lie in a different component or domain than the observed effect. In practice, the multi-domain, multimodal nature of IoT system components has led to a fragmented approach to reliability, dominated by domain-specific, often component-centric approaches that are inadequate for capturing failure–cascade dynamics and interface failures [12]. Simply put, IoT system reliability cannot necessarily be derived from the reliability of individual components.

Previous work has addressed the challenges associated with IoT systems and reliability from a “design for reliability” (DfR) perspective by developing a common understanding of reliability across system domains [13]. However, while DfR approaches mitigate reliability concerns in system design, they do not necessarily improve the handling of failures in operational systems. The same challenges seen in the DfR domain for IoT systems are mirrored in the field, where the complex system dynamics, cascading failure scenarios, and interface failures make detecting, diagnosing, and mitigating failures a complicated venture.

### 2.2. Taxonomy of FDD Methods

Fault detection and diagnosis methods for time-series telemetry can be grouped into four broad families, each with characteristic strengths and deployment tradeoffs [14]. Understanding this landscape is necessary to motivate the methodological choices in this work, since IoT FDD is rarely a matter of which family is universally best, but rather of which family best fits the data, labeling, and operational constraints of a given deployment. In IoT-specific settings, practical detectors often emphasize deployability, for example, correlation-change feature selection [15] and KPI-driven detection in cyber-physical systems [16].

1. **Statistical and rule-based detectors** apply fixed thresholds, control charts, or pointwise statistical tests (e.g., z-score, CUSUM, and Shewhart charts) to individual signals. These methods are computationally trivial, require no training data, and produce interpretable alerts, which is why they remain the default in many embedded and IoT systems [9,10]. For systems where reliability requirements drive the design from the hardware layer upward, these conventional detectors are necessary but rarely sufficient [11,12]. Their limitations are also well known [1,2], namely:
  - Samples are assumed and treated independently;
  - Insensitive to gradual drift or temporally structured deviations;
  - Manual threshold tuning that does not generalize across devices or operating conditions is required.
2. **Supervised classification methods** learn a mapping from labeled fault examples to fault categories using algorithms ranging from support vector machines and random forests to deep neural networks. When sufficient labeled data exists, these methods can achieve high accuracy and fine-grained fault categorization. However, supervised classifiers are practically constrained, particularly for IoT systems, by the requirement for labeled data. Deployed IoT systems rarely accumulate the volume and variety of labeled fault events necessary to train competitive supervised models, and the fault surface is often unknown at the time of deployment [3,4]. Industrial fault diagnosis reviews consistently identify label scarcity as the primary obstacle to deploying supervised learning in real-world monitoring contexts [3].
3. **Unsupervised representation learning methods**, such as autoencoders, variational autoencoders, generative adversarial networks, and self-supervised transformers, learn a compressed representation of normal behavior and flag inputs that the model reconstructs poorly or assigns low likelihood. These methods are powerful for high-dimensional or visually complex telemetry. Still, they introduce significant deployment overhead: nontrivial architecture and hyperparameter selection, GPU training requirements, opaque anomaly scores that are difficult to interpret, and sensitivity to distribution shifts within the training window [14]. Recent comparative studies of multivariate time-series classification note that no single representation-learning approach dominates across datasets, and that simpler baselines often remain competitive despite their architectural simplicity [17].
4. **Similarity-based methods** compare new observations to a library of reference examples using a distance or similarity measure, flagging observations that are dissimilar to all references. This family includes nearest-neighbor methods, kernel density estimators, and shape-matching methods, such as Dynamic Time Warping. Similarity-based methods are label-free (the reference library requires only assumed-healthy data), computationally efficient, and naturally interpretable; the closest reference example provides a direct explanation for why an observation was flagged. The main limitations are sensitivity to feature scaling, the need to manage the reference library size, and reduced effectiveness when faults preserve the overall shape of the signal.

The DTW-based reference-library method developed in this work is a similarity-based approach. It is well-suited to the IoT FDD setting precisely because it requires no labeled fault data, supports interpretable per-channel and per-layer attribution, handles temporal misalignment naturally (a common feature of IoT telemetry with variable sampling rates), and can be deployed without GPU infrastructure or extensive model selection. These properties make DTW reference-library scoring a strong candidate for early-stage, label-scarce deployments that this paper targets, while leaving room for hybrid pipelines that combine DTW with statistical or learning-based detectors when warranted by the fault profile.

### 2.3. Dynamic Time Warping in Modern Time-Series Analytics

DTW has been a core method for assessing similarity in time-series data since its introduction in 1978, and has found wide application across domains [7,8]. Originally developed for speech recognition, DTW is a popular method for general time-series analytics that aligns sequences under local time distortions and supports intuitive interpretations through warping paths [9]. Recent work continues to extend DTW for practical constraints and domain structure, including constrained bands, feature-aware distances, and non-Euclidean variants, such as spherical DTW [18,19].

The DTW-based pipeline applied to multivariate IoT telemetry in this work follows a common high-level structure. The continuous telemetry stream from a device is first divided into overlapping fixed-length windows called episodes, each capturing a short segment of behavior across all available sensor and derived signals. Within each episode, the individual signals, temperature, voltage, communication health proxies, and derived behavioral features are referred to as channels, which are grouped into layers corresponding to the hardware, communication, and software/firmware aspects of the device. During an initial training period assumed to be free of significant faults, a reference library is constructed by storing the per-channel time series from many normal episodes. At evaluation time, each new episode is compared against the library using DTW, yielding a distance score for each channel that quantifies how dissimilar the episode is to its closest normal template. These per-channel distances are aggregated into layer- and episode-level anomaly scores, and episodes with scores exceeding a learned threshold are flagged as anomalous. The retained per-channel and per-layer distances also support attribution, identifying which subsystem and which signals contributed most to the deviation. Section 3 and Section 4 formalize this pipeline and describe the design choices required to make it operational.

In fault diagnosis and prognostics, DTW and its variants are used for pattern matching under variable operating conditions (e.g., speed variation) [20,21] for similarity-based anomaly detection and as a preprocessing step in hybrid pipelines. Examples include Mahalanobis distance-based DTW for fault detection [22], adaptive decomposition and DTW for gearbox analysis [23], current-aided DTW for planetary gearbox faults under time-varying speeds [24], and DTW-based anomaly detection in predictive maintenance [25]. DTW is also used outside of rotating machinery, including distribution-system fault classification pipelines that use fast DTW with time-frequency transforms [26], vehicle analytics (i.e., Internet of Vehicles, IoV) that use multivariate DTW [27], and collaborative real-time fault diagnosis frameworks in transportation infrastructure [28]. Recent hybrid approaches have also combined DTW with deep learning architectures for multivariate time-series classification [29]. These studies reinforce a pragmatic view: DTW is a strong building block for rapid, label-light deployments when faults manifest as temporally structured deviations in signal shape.

## 3. Motivation and Problem Formulation

Unlike the curated benchmark datasets commonly used to validate FDD approaches, operational IoT systems typically lack reliable fault labels. Faults may be rare, intermittent, or masked by operational changes. Even when a failure event is known, the exact onset time and affected signals may be uncertain. Intermittent fault recognition has, therefore, become a priority topic, with emphasis on feature engineering, temporal localization, and weakly supervised evaluation protocols [6].

For multivariate time series, algorithm performance and robustness can vary substantially across domains. Extensive comparative studies in time series classification show that no single model dominates across all datasets and problem settings, motivating the need for practitioner guidance and model selection strategies rather than one-size-fits-all solutions [15].

This section formalizes the episodic, layer-based, system-level fault detection architecture for IoT telemetry data. The formulation is intentionally practitioner-oriented: it emphasizes deployability, particularly in label-scarce environments, and produces diagnostic outputs that map to engineering-domain responsibilities (hardware, communications, and software/firmware). An IoT device produces a multivariate time series $X\left(t\right)=\left[x_{1}\left(t\right),x_{2}\left(t\right),\ldots ,x_{C}\left(t\right)\right]$, where channels $x_{i}$ may include raw sensor measurements, communication indicators, and derived telemetry features. Telemetry is resampled onto a uniform grid with a sampling period Δ. After resampling, each channel becomes a discrete-time sequence $x_{c}(k)$, where k = 0, 1, 2, … is an integer counter that identifies successive samples on the uniform grid. The k-th sample of channel c corresponds to the wall-clock time $t_{0}+k∆t$, where $t_{0}$ is the start of the resampled record. All subsequent processing operates on this discrete-time representation.

*Episodes* are generated by segmenting the time series into overlapping windows, each with length T and stride S. Each episode contains channel sequences $E_{i}\left[c\right]=\left\{x_{c}\left(k\right)\right\}$ for k within the episode interval. These episodes capture system behavioral patterns, such as duty-cycle changes, drift, and dropout, which unfold over minutes to hours and are often missed by pointwise detectors.

*Channels* are grouped into system layers $\ell \in \left\{HW,\;COM,\;SW\right\}$, representing hardware sensing, communication/data integrity, and software/firmware (SW/FW) behavioral proxies, respectively. Layering supports diagnosis by aggregating evidence at the level of likely root-cause domains. Importantly, layers are an analytical construct and do not require full-stack instrumentation; for example, SW/FW features can be approximated using rolling variance, change rate, and energy derived from raw sensor data.

A *reference library* L is built from an initial training interval that is predominantly healthy (but not perfectly curated). Within this interval, representative normal episode templates are stored for each channel. The library supports similarity-based scoring without labeled fault classes.

Evaluation involves computing a per-channel distance to the library for each episode $E_{i}$, using constrained DTW, retaining the best-match distance. The channel distances are aggregated into layer scores, which are then combined into an overall episode anomaly score $S\left(E_{i}\right)$. The episodes are flagged as anomalous when $S\left(E_{i}\right)$ exceeds a threshold τ (tau) learned from training-normal scores. Attribution is achieved by ranking layers and channels by their DTW distances, thereby facilitating diagnosis and triage.

**Figure 2:**
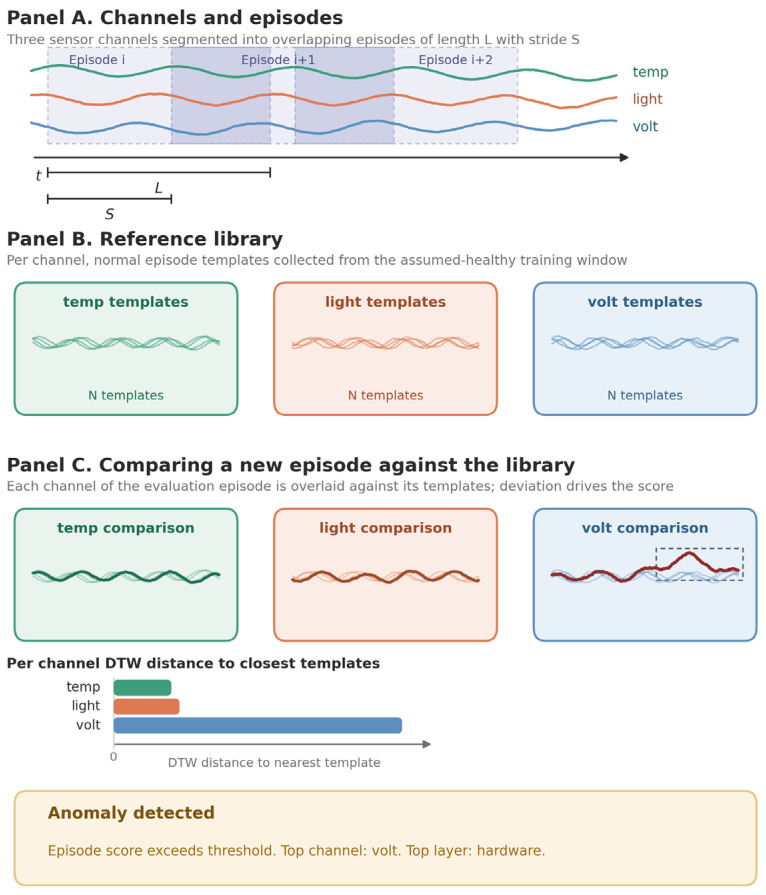
Episode- and layer-based DTW comparison for IoT fault detection. (**A**) Multivariate telemetry (in this case, temperature, light, and voltage) is segmented into overlapping episodes of length L and stride S, with each channel contributing a separate signal trace within every episode. (**B**) A reference library is built from the assumed-healthy training window as a per-channel collection of N normal episode templates. (**C**) Each channel of an evaluation episode (solid trace) is compared with its template (faded trace) using the constrained DTW. The voltage channel deviates from the normal envelope (red-shaded region), resulting in a high per-channel distance and triggering an anomaly detection, with voltage as the top-contributing channel.

The objective of this methodology is to process unlabeled IoT telemetry to produce: (i) an episode-level anomaly score timeline, (ii) a binary anomaly decision using an operational threshold, and (iii) interpretable attributions to layers and channels that enable engineers to localize faults across the system stack. See Figure 2 for a visual representation of this approach to IoT FDD.

## 4. Method: DTW-Based System-Level Fault Detection in IoT Telemetry

This section details the end-to-end DTW-based FDD pipeline, both in general and as used in the demonstration. It highlights the practical tuning parameters that convert an academic similarity measure into a deployable IoT monitoring baseline. The method follows the episode and layer abstractions defined in Section 3: telemetry is segmented into overlapping episodes, channels are mapped into interpretable layers, and anomalies are detected by comparing each episode to a library of regular reference episodes.

### 4.1. Dataset and Experimental Design

The approach is demonstrated using the Intel Berkeley Research Lab sensor dataset [30], a multivariate IoT telemetry dataset containing approximately 2.3 million time-stamped sensor readings collected over 34 days from 54 wireless motes. Each reading includes temperature, humidity, light, and battery voltage. The dataset is representative of real IoT monitoring constraints: it is large, multi-sensor, and unlabeled with respect to faults and root causes. Because the method scores the temporal shape of telemetry rather than sensor- or platform-specific characteristics, its applicability is independent of sensor generation and hardware.

For the demonstration, telemetry from a single representative mote (mote 31, selected for record completeness and temporal coverage across 32 days) is used. This single-device focus aligns with the system-level formulation in Section 3, where each IoT device is monitored independently across its hardware, communication, and software/firmware layers. Mote 31’s battery voltage drops over the deployment (from 2.70 V to 2.19 V), and from day 24 onward, its temperature and humidity sensors return corrupted values (temperature saturates near 122 °C and humidity goes negative). To avoid contaminating the normal class with this real end-of-life degradation, both the reference library and the synthetic-fault evaluation are placed inside the verified-healthy span: days 0 to 18 train the reference library, and days 18 to 24 (approximately six days, all pre-corruption) form the evaluation interval. The post-day-24 degradation is itself a genuine, unlabeled hardware fault; scored against the healthy library, it produces a sustained, unambiguous anomaly, a real-fault demonstration alongside the synthetic injection.

Because the dataset does not include ground-truth fault annotations, evaluation is performed via synthetic fault injection on the held-out evaluation interval. Four fault types are injected at known times: stuck-at (constant-value), bias shift, gradual drift, and intermittent spikes. Each fault affects a single sensor channel for 2 h, and the faults are spaced to avoid temporal overlap. The system is trained label-free by building a reference library from the training window; injected faults provide ground truth for quantifying detection performance. Fault injection parameters (magnitude, slope, and amplitude) are specified per fault type to ensure reproducibility. This use of injection provides traceable ground truth for computing precision and recall and is a controlled validation step rather than a model of the full operational fault surface. The injected types correspond to documented physical failure modes: stuck-at to sensor or ADC latch-up and transport-layer freezes; bias shift to calibration drift or reference-voltage offset; gradual drift to thermal aging or sensor fouling; and intermittent spikes to electromagnetic interference or supply transients.

### 4.2. Preprocessing and Normalization

Raw records are parsed into a time-indexed multivariate series, filtered to the target mote, sorted, and deduplicated. To support DTW comparisons across time, the series is resampled to a uniform 1-min grid. Within each sensor channel, missing values are forward-filled, then backward-filled, to avoid dropping entire episodes. Finally, each channel is robustly normalized using median and median absolute deviation (MAD) scaling to reduce sensitivity to outliers and scale differences across sensors. Channels with zero MAD (constant values) fall back to standard-deviation scaling.

### 4.3. Layered Feature Construction

To align with system-level IoT diagnosis, channels are organized into three layers, each producing a set of features that serve as DTW comparison targets:

- Hardware (HW): The four primary sensor channels (temperature, humidity, light, and voltage) after robust normalization. These form the direct observables of the physical system.
- Communication (COM): Health proxies derived from the telemetry stream. In the implementation, these include per-channel stuck-value rates (rolling detection of repeated identical readings, which may indicate transport-layer freezes) and low-variance indicators (rolling windows, where variance drops below the 10th percentile of the channel’s variance distribution, suggesting irregular sampling or data staleness). These proxies approximate missingness and gap behavior without requiring packet-level instrumentation.
- Software/Firmware (SW/FW): Derived key performance indicators (KPIs) that capture operational dynamics not directly modeled by physics-of-failure. For each sensor channel, five rolling-window features are computed: rolling variance, absolute change rate, duty cycle (fraction of time above median), signal energy (sum of squared values), and zero-crossing rate. These features are sensitive to behavioral regime changes, such as firmware duty-cycle shifts, workload transitions, or sensor degradation patterns, that alter signal dynamics rather than magnitude.

This layer mapping produces 4 HW features, 8 COM features, and 20 SW/FW features per episode, allowing the method to attribute anomalous behavior not only to a single sensor channel but also to a subsystem abstraction. In practice, this is critical for emergent faults where symptoms are distributed across layers.

### 4.4. Episode Segmentation

The preprocessed multivariate series is segmented into overlapping episodes using a sliding window of length L minutes and a stride of S minutes. Episode length is a primary operational tuning parameter: shorter episodes reduce detection delay but are more sensitive to noise; longer episodes increase stability and improve precision when normal behavior exhibits daily or workload-driven structure. Episodes that lack complete data across all layers (e.g., due to sensor gaps at the boundaries of the data record) are discarded. In the tuned configuration, L=60 min and S=20 min provided the best overall performance on the injected-fault evaluation.

### 4.5. Reference Library of Normal Templates

The reference library encodes the normal behavior for each channel and serves as the comparison set for all DTW scoring in Section 4.6. Because the design of the library directly affects both detection performance and computational cost, its structure and construction are described in detail.

A template is a single, normalized time series representing a channel’s values across a single training-window episode. For an episode of length L sampled at 1-min resolution, each template is a vector of L floating-point values for one channel, for example, the temperature trace from one specific 60-min window in the training period. Templates are stored per channel rather than as multivariate episode snapshots, so each training episode contributes one template to each of the 32 channel-specific template sets (4 HW, 8 COM, and 20 SW/FW). This per-channel organization is what enables the per-channel attribution reported in Section 4.7.1: an evaluation episode can deviate strongly on one channel while remaining nominal on others, and the library structure preserves that distinction throughout scoring.

The library is constructed in three steps. First, all training-window episodes are enumerated, and the time series for each channel is extracted from each episode. Second, candidate templates are validated: those containing NaN or non-finite values, or fewer than two valid samples, are discarded. Third, if the number of valid candidates for a channel exceeds the configurable cap N (set to 15 in the tuned configuration), the templates are selected via uniform subsampling of the original temporal ordering. Uniform subsampling is preferred over random selection because it provides deterministic, reproducible coverage of any diurnal or workload-driven structure present in the training period, which is desirable for both reproducibility and the preservation of the temporal diversity of the resulting template set. After construction, the library is represented as a mapping from channel names to lists of templates, accompanied by metadata that records the source episode count, training-window timestamps, and per-channel template counts.

The template cap serves two purposes. The first is computational: DTW scoring time scales linearly with the number of templates per channel, and an uncapped library built from a 25-day training window with 60-min episodes and a 20-min stride would contain approximately 1,800 templates per channel, making library sizes prohibitive for repeated scoring over long evaluation intervals. Capping at 15 templates per channel reduces this by two orders of magnitude. The second purpose is regularization: a smaller, temporally diverse template set generalizes better than a larger set populated with near-duplicate observations of the same recurring cycle.

It is important to note what the library does not contain. No fault templates, no labeled examples, and no anomaly information of any kind are used in its construction; the library is built exclusively from the assumed-healthy training window. This is the structural basis for the label-free property of the method. A consequence of this design is that the library is sensitive to training-window contamination: any undetected faults present in the training period will be encoded as normal behavior, and subsequent similar faults may evade detection. This limitation, together with mitigation strategies based on outlier-aware template selection and post hoc library refinement, is discussed in Section 6.2.

### 4.6. Constrained DTW Scoring

For each evaluation episode, DTW distances are computed between the episode’s channel time series and every template in the reference library for that channel. The implementation uses constrained DTW with a Sakoe–Chiba band [7,9] to bound the warping path and reduce both runtime and the number of pathological alignments. Scoring proceeds in three stages:

1. Per-channel best-match distance: For each channel, the DTW distances to all templates are computed, and the best-match score is taken as a low percentile (5th percentile) of these distances rather than the strict minimum, improving robustness to individual poorly matched templates.
2. Layer aggregation: Channel scores are grouped by layer (HW, COM, and SW/FW) and averaged within each layer to produce per-layer scores.
3. Standardization: Each channel’s best-match DTW distance is standardized against its own training-normal distribution (a robust z-score using the training median and MAD), placing all channels on a comparable scale so the informative shape features (rolling variance, change rate, zero-crossing rate, and duty cycle) are not overwhelmed by the high-magnitude signal-energy features. The episode score is the mean of these standardized distances over the 28 non-energy channels; that is, all channels except the four per-sensor signal-energy features (Section 4.3), excluding the energy features, which carry little shape information, gives the strongest separation (Section 5.2)

This per-channel standardization is more effective than restricting scoring to the hardware layer. Under standardized scoring, the hardware-only channel set is the weakest (AUROC 0.63) because the raw-level channels are noisier and drift-sensitive, while the standardized non-energy set is the strongest (AUROC 0.96).

### 4.7. Robust Threshold Learning and Temporal Validation

A key deployment challenge is converting DTW scores into actionable alarms without labels. A simple percentile threshold on training scores can be overly sensitive when the training set contains regime changes or when derived features dominate the score distribution. Therefore, a robust, multi-method thresholding strategy is used, with candidate thresholds computed using:

(i) A high-percentile rule;
(ii) An interquartile range (IQR) rule;
(iii) A standard deviation (sigma) rule.

The operational threshold is learned from training-normal scores only, combining the percentile, IQR, and sigma candidates above. An alert-rate budget sets the operating point, a chosen percentile of the training-normal score distribution, which makes the expected false-alarm rate explicit and uses no evaluation-set information.

To further reduce spurious alarms, temporal validation is applied through a two-layer filter. First, an episode is flagged only if its score exceeds the threshold by a minimum multiplicative factor α (the score-threshold multiplier). Second, an anomaly is asserted only when at least M episodes within a local window (the current episode and its immediate neighbors) exceed 80% of the threshold, requiring local persistence of the anomalous signal. In the selected configuration, M=2 and α=1.4 suppress isolated excursions while maintaining an acceptable detection delay.

#### 4.7.1. Layer and Channel Attribution

Beyond binary anomaly flags, the method supports diagnosis by retaining per-channel DTW distances from the best-matching template. Channels are ranked by distance to identify the dominant contributors, and distances are aggregated by layer to identify which subsystem abstraction (HW, COM, or SW/FW) most explains the deviation. In the tuned configuration, per-channel robust-z standardization and the exclusion of high-magnitude signal-energy features (Section 4.6) prevent high-variance-derived features from overwhelming the anomaly score and attribution. The top-contributing layer and the top three contributing channels are reported for each detection, providing an actionable starting point for fault triage.

#### 4.7.2. Parameter Tuning for Deployment

While DTW itself is a fixed similarity measure, IoT FDD performance depends on a small set of operational parameters. In deployment, these are set based on operational knowledge and label-free criteria rather than on evaluation data: episode length and stride from the monitoring cadence and the expected fault timescale; the threshold from an alert-rate budget on the training-normal score distribution; and the temporal-validation settings from the desired trade-off between false-alarm suppression and detection delay. Section 5.2 shows that the method is robust to these choices, so precise tuning is not required. The parameters with the most influence are:

- Episode parameters: episode length L and stride S (controls noise sensitivity and delay).
- Threshold sensitivity: percentile *p*, IQR multiplier *k*, and sigma multiplier *σ* (controls false positives).
- Temporal validation: minimum consecutive anomalies *M* and score multiplier *α* (filters transient excursions).
- Channel standardization and selection: per-channel robust-z scaling of DTW distances and the set of channels scored (controls feature dominance and noise).

**Figure 3:**
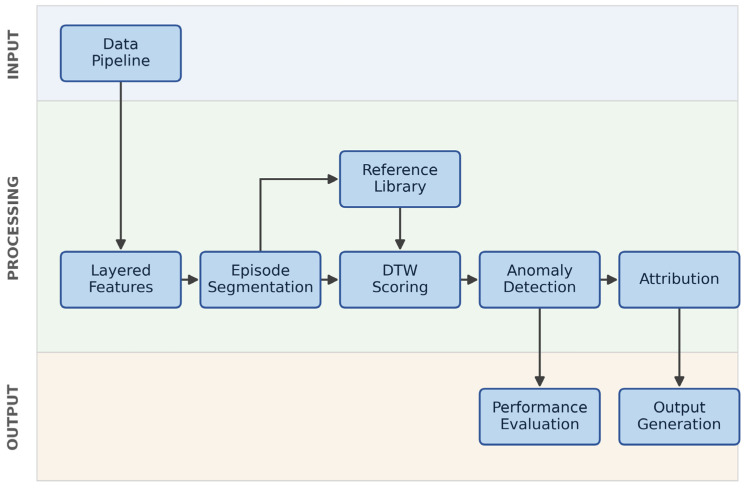
DTW-based IoT fault detection system architecture (episode and layer abstractions with DTW scoring, attribution, and evaluation).

The overall system architecture for this DTW-based FDD approach is shown in Figure 3. Section 5 reports the method’s detection performance and its sensitivity to these settings.

## 5. Results and Findings

This section evaluates the method on the verified-healthy window, using synthetic fault injection to provide ground truth. It reports the configuration and its rationale (Section 5.1), the sensitivity to the main parameters (Section 5.2), the operating-point behavior and confusion matrix (Section 5.3), the per-fault-type separability and detection delay (Section 5.4), the anomaly score timeline (Section 5.5), and a quantitative summary with layer attribution (Section 5.6).

### 5.1. Configuration and Design Rationale

The configuration used throughout is as follows: episodes of length L = 60 min at a stride of S = 20 min, a reference library of up to 15 templates per channel, per-channel robust-z standardization of DTW distances, an episode score equal to the mean standardized distance over the non-energy channels, and an alert threshold set from the training-normal score distribution. Two design choices are central. First, because raw DTW distances differ in scale across channels, a few high-variance channels would otherwise dominate the episode score; per-channel standardization and exclusion of the high-magnitude energy features place the informative shape features on an equal footing. Second, because the device’s sensors become corrupted near the end of life, the reference library and the evaluation are confined to the verified-healthy window (Section 4.1). Section 5.2 reports how performance varies with these parameters.

### 5.2. Parameter Sensitivity

**Figure 4:**
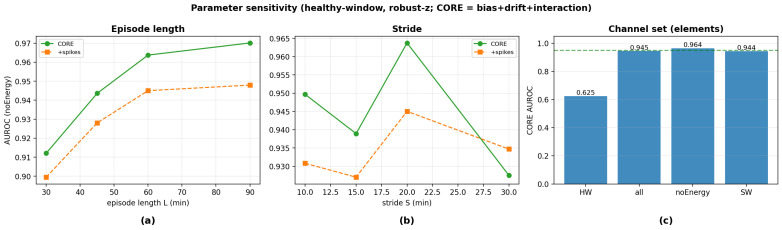
Parameter sensitivity. (**a**) AUROC versus episode length L and (**b**) AUROC versus stride S, with the solid line for CORE and the dashed line adding the spikes class. (**c**) CORE AUROC by channel set; the dashed horizontal line marks the AUROC obtained using all 32 channels (0.945), which the non-energy set (0.964) exceeds and the hardware-only set (0.625) falls well below.

To characterize the method’s sensitivity to its main parameters, we evaluated episode length, stride, and channel set on the healthy-window evaluation, using the threshold-independent AUROC over the DTW-appropriate fault classes (bias, drift, and interaction). Table 1 reports the results, and Figure 4 visualizes them.

### 5.3. Operating Point and Confusion Matrix

The continuous score is converted to a binary alarm by a single, label-free knob: a percentile of the training-normal score distribution sets the design false-alarm rate. At an alert budget of about 5%, the detector is balanced (precision and recall near 0.85); raising the budget trades alarms for recall (Appendix Table A1). Because the score’s separability is high (AUROC 0.97), the operating point can be chosen freely to suit the deployment’s alarm tolerance, α=1.4.

At the 95th-percentile budget, the episode-level operating point yields the confusion matrix in Table 2. The episode-level false negatives are predominantly low-overlap boundary windows (median 17% fault overlap versus 50% for detected episodes); every injected fault is still detected, giving 100% fault-level recall, so no fault is missed at any of these operating points.

### 5.4. Fault-Type Detection and Detection Delay

**Figure 5:**
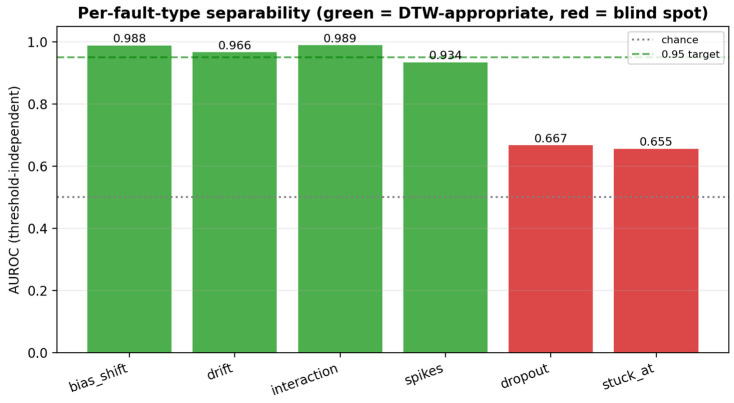
Per-fault-type separability.

**Figure 6:**
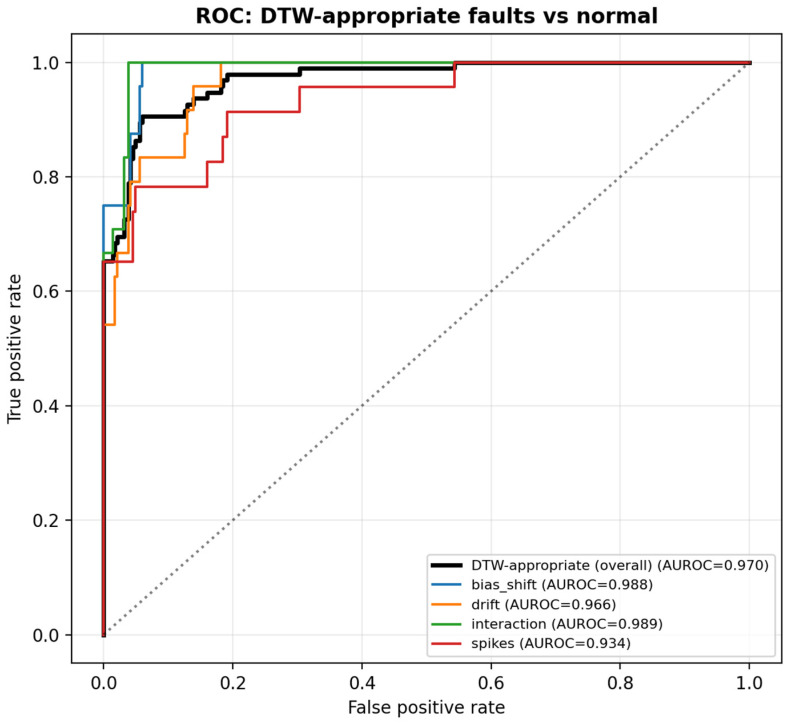
ROC curves for DTW-appropriate faults versus normal episodes (bold black = overall, AUROC = 0.97; colored = per fault type, AUROC 0.93–0.99). The dotted diagonal is the chance baseline (AUROC = 0.5); curves nearer the upper-left corner indicate better separation.

Per-fault-type separability shows where DTW is the right tool. For the temporally structured faults DTW is suited to, the threshold-independent AUROC is high (bias 0.99, interaction 0.99, drift 0.97, and spikes 0.93), with 100% fault-level detection. Constant-value (stuck-at) and missing-data (dropout) faults fall to an AUROC of about 0.66, near chance; they are structurally outside DTW’s shape-matching scope and are routed to complementary detectors (a variance or range check for stuck-at; a missingness monitor for dropout). This reflects DTW’s scope for shape matching rather than a parameter choice. Table 3, Figure 5 and Figure 6 report the per-type results.

Because the detector operates on episodes with temporal validation, alarms incur a bounded detection delay; across the DTW-appropriate faults, the mean delay is 21 min (Table 3), well within the sub-60-min target and bounded by the episode stride and length.

### 5.5. Anomaly Score Timeline

**Figure 7:**
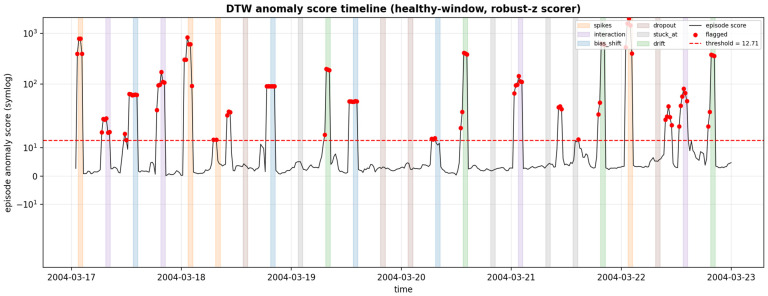
The DTW anomaly score timeline (verified-healthy window, robust-z scorer). The dashed line is the alert-rate threshold.

Figure 7 shows the anomaly score timeline over the verified-healthy evaluation window, with the injected-fault intervals shaded by type. The score remains in a tight nominal band and rises sharply during the DTW-appropriate fault intervals. In contrast, the stuck-at and dropout intervals stay near the nominal envelope, consistent with their out-of-scope status.

The evaluation interval is the verified-healthy window (days 18 to 24, approximately six days before the mote’s end-of-life sensor corruption). The DTW anomaly score timeline (Figure 7) shows a well-defined nominal regime with sparse, high-contrast excursions that align with the DTW-appropriate fault intervals. In contrast, stuck-at and dropout intervals remain near the nominal envelope, consistent with their out-of-scope status.

### 5.6. Quantitative Performance Summary

This section summarizes the quantitative detection performance on the verified-healthy evaluation: the threshold-independent AUROC over the DTW-appropriate fault classes, the per-fault-type separability, and the episode-level operating points across alert budgets.

#### 5.6.1. Summary of Detection Performance

Table 4 summarizes the detection performance. The headline is the threshold-independent AUROC of 0.97 on the DTW-appropriate fault classes, computed under a predefined configuration and a threshold learned from training-normal scores within the verified-healthy window; it is not influenced by threshold or operating-point choices. Fault-level detection is 100% (16/16), with a mean delay of 21 min.

The threshold-independent AUROC of 0.97 confirms strong separation between normal and DTW-appropriate fault episodes. The operating-point precision and recall across alert budgets are reported in Appendix Table A1.

#### 5.6.2. Layer Attribution Behavior

Under the tuned configuration, layer attribution remains dominated by software/firmware (SW/FW) features, particularly rolling energy and variability metrics derived from temperature and light signals. This behavior reflects the fact that injected faults primarily alter behavioral dynamics rather than raw sensor magnitudes. Under the standardized scorer, the hardware-only channel set is the weakest on the synthetic benchmark (AUROC 0.63) because the mote’s real battery degradation dominates it; the hardware layer, nonetheless, remains essential for detecting genuine hardware faults, as the post-day-24 corruption demonstrates.

Importantly, the standardized scorer produces stable attribution results without overwhelming false positives. Nevertheless, these results highlight a practical consideration: per-layer or per-feature normalization after feature computation can further improve the balance of attributions when feature families operate on different numeric scales.

### 5.7. Explainability: DTW Alignments and Localized Distance Profiles

**Figure 8:**
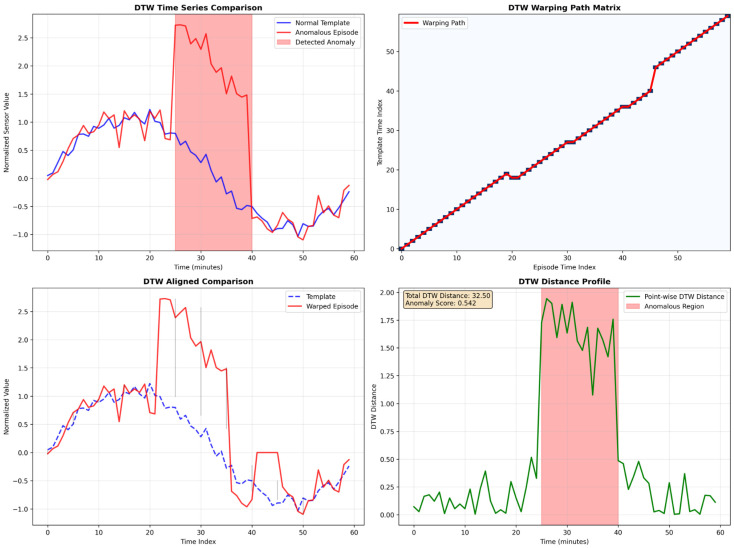
DTW alignment example illustrating the time-series divergence (**Top and Bottom Left**), constrained warping path (**Top Right**), and a localized DTW distance profile (**Bottom Right**) for anomaly explanation.

DTW provides interpretability beyond scalar anomaly scores. By inspecting the warping path and pointwise distance profile, practitioners can localize where an episode diverges from its closest standard template and determine whether the deviation is consistent with drift, bias, burst, or dropout. Figure 8 shows a representative alignment: the anomalous episode departs from the standard template within the detected anomaly region, while the warping path remains well-behaved due to band constraints.

## 6. Discussion: Practical Impact, Limitations, and Deployment Guidance

The results in Section 5 demonstrate that a DTW-based episode-scoring method can achieve strong detection performance (AUROC 0.97 on the DTW-appropriate fault classes) on IoT telemetry data under label-scarce conditions, while providing interpretable attributions across system layers. The path from a naive deployment to this result depends on per-channel standardization, exclusion of high-magnitude energy features, and a verified-healthy reference window, as detailed below.

### 6.1. Value of DTW in IoT FDD

The primary strength of DTW in IoT fault detection lies in its ability to compare behavioral shape under temporal misalignment. This property is well matched to IoT telemetry, where equivalent system behaviors may occur with phase shifts, variable sampling rates, or workload-dependent timing. In the experiments presented, DTW reliably detected drift-, bias-, and spike-type faults without requiring labeled fault data for training, confirming its suitability for early-stage and evolving deployments.

A second key advantage is label-free deployment. Reference-library scoring can be trained on an assumed-healthy period, which aligns with common IoT operational realities in which comprehensive fault catalogs are unavailable at deployment time. DTW naturally incorporates priors from simulation and testing by allowing known nominal regimes to be represented as templates. DTW also offers practical interpretability benefits. Warping paths and template alignments provide human-interpretable explanations for why an episode is flagged as anomalous, enabling faster triage than opaque anomaly scores produced by many representation-learning methods. Finally, the episode-and-layer formulation enables more robust system-level reasoning. By aggregating DTW distances across the HW, COM, and SW/FW feature groups, the method supports fault localization across system domains. This mapping aligns well with engineering ownership boundaries and reduces time to root-cause narrowing.

Compared to alternative unsupervised approaches, the DTW reference-library method occupies a distinct niche. Pointwise statistical detectors (e.g., z-score thresholding or control charts) can identify isolated exceedances but do not capture sustained behavioral deviations that unfold over an episode. They are, therefore, prone to miss gradual drift or cross-channel interaction faults of the kind demonstrated here. Reconstruction-based methods, such as autoencoders, learn a compressed representation of normal behavior and flag episodes with high reconstruction error. Still, they require nontrivial architecture selection and training, and their anomaly scores are generally less interpretable than DTW alignment paths. Isolation Forest and similar tree-based anomaly detectors operate on feature vectors. They can be effective for tabular anomaly detection, but they discard the temporal ordering within an episode. They thus cannot exploit the shape-matching capability that is central to DTW’s sensitivity to drift and phase-shift faults. The DTW approach trades modeling flexibility for deployment speed, interpretability, and minimal training assumptions, properties particularly valuable in the early-deployment and label-scarce settings this paper targets. Boundary-based detectors, such as One-Class SVM, share the feature-vector limitation noted above: they characterize a normal region in feature space without modeling intra-episode temporal structure. Rather than ranking methods in the abstract, Table 5 evaluates the principal FDD families against the specific deployment requirements of this work. The episode-and-layer DTW approach is the only configuration that satisfies all of them and provides both episode-scale temporal sensitivity and engineering-aligned cross-layer attribution.

### 6.2. DTW Challenges and Potential Solutions

DTW is not universally effective, and its limitations are important to consider for proper deployment. The principal challenges observed in the experiments with DTW architectures include:

1. Computational cost scales with episode length and the number of reference templates. Constrained DTW with a Sakoe–Chiba band of width w has time complexity O(L·w) per template comparison, so the per-episode scoring cost is O(C · N · L · w), where C is the number of channels and N is the per-channel template cap. In the tuned configuration (C = 32, N = 15, L = 60, and w = 6), this corresponds to roughly 173,000 cell computations per episode, with an empirical scoring time of approximately 11 s per episode on a consumer-grade workstation. Doubling the episode length quadruples the per-episode cost when the bandwidth is scaled proportionally, while doubling the template cap or the number of channels scales the cost linearly. High-rate telemetry or vast libraries may require approximate DTW variants (e.g., FastDTW) [31], scalable exact-search techniques [32], or alternative feature-based detectors. The memory footprint is correspondingly small: the reference library stores C × N × L values (here, 32 × 15 × 60, or about 28,800 samples), on the order of a few hundred kilobytes, and scoring requires only a single L-by-W cost band in working memory per comparison. The method is designed to run on an edge gateway or fleet backend that ingests device telemetry rather than in situ on the sensor node, and it scores complete episodes spanning minutes to hours rather than operating at a sample rate. Under this model, on-node compute and memory limits are not the binding constraints. The relevant timing question is whether scoring keeps pace with the monitoring cadence. At approximately 11 s per episode, against a 20-min stride and a sub-60-min detection-delay target, it does so with a substantial margin. The small footprint also makes gateway-class edge deployment feasible where desired; on-device deployment on resource-constrained sensor nodes and benchmarking on specific embedded targets are left to future work.
2. DTW is sensitive to feature scaling and feature dominance. In the reported experiments, the derived SW/FW energy features dominated both scoring and attribution when all layers were weighted equally, due to their larger numeric scale. The tuned configuration mitigates this by standardizing each channel’s DTW distance to its own training-normal distribution and excluding high-magnitude signal-energy features (Section 4.6); the resulting non-energy channel set yields the strongest separation (Section 5.2). As a complementary or alternative strategy, practitioners should consider normalizing features after computation or applying per-layer weighting, and should validate that attribution aligns with engineering expectations rather than artifactually reflecting feature magnitude.
3. Threshold calibration is nontrivial. Percentile-based thresholds computed on training scores can become unstable when training episodes are highly similar to reference templates (e.g., when identical or near-identical episodes are compared). Practical mitigations include:
  - Scoring training episodes against a disjoint template subset;
  - Leave-one-out scoring during threshold estimation;
  - Selecting thresholds based on an alert-rate budget rather than a fixed percentile.
4. DTW is a shape-matching method and is structurally blind to flat or missing-signal faults: constant-value (stuck-at) and dropout faults score near chance (AUROC about 0.66). This is a scope boundary rather than a tuning deficiency, and such faults are routed to complementary detectors: a variance or range check for stuck-at, and a missingness monitor for dropout, which run alongside DTW at negligible cost. DTW is, therefore, best deployed as the first-line detector for temporally-structured faults within a small ensemble.

### 6.3. Deployment and Tuning Guidelines

The experiments highlight that tuning and workflow design matter as much as the DTW algorithm itself. The parameter sensitivity analysis (Section 5.2) showed that the threshold-independent AUROC on DTW-appropriate faults varies smoothly with episode length, stride, and channel set, with the standardized non-energy channel set as the dominant lever, demonstrating that disciplined feature standardization and selection, rather than algorithmic complexity, drive performance.

1. Start conservatively. Use a high-threshold percentile and validate false positives against a known-normal window before lowering sensitivity.
2. Tune episode length deliberately. Longer episodes tend to improve precision by emphasizing sustained behavioral change, while shorter episodes reduce detection delay but increase noise sensitivity.
3. Use robust thresholding. Combine percentile-based thresholds with IQR- or sigma-based checks and select a median threshold to reduce sensitivity to regime shifts.
4. Add temporal validation. Require consecutive anomalous episodes before alerting to suppress isolated spikes and improve operational stability.
5. Preserve attribution outputs. Layer and channel rankings should be retained and reviewed as part of the diagnostic workflow; they are essential for root-cause analysis and iterative feature refinement.

DTW is best viewed as a first-line detector in an IoT fault-detection pipeline. It provides immediate value under sparse labeling and offers interpretable insights that help build organizational understanding of failure modes. As labeled data accumulates, DTW-based scoring can inform model selection and hybrid supervised approaches, enabling a measured transition to more complex methods with clear return on investment.

## 7. Conclusions

This paper presented a practitioner-oriented DTW-based method for system-level fault detection and diagnosis in IoT telemetry under a label-scarce setting. Telemetry was modeled as overlapping episodes and organized into interpretable hardware, communication, and software/firmware layers, yielding a deployable, label-free monitoring approach that supports both detection and triage. The contribution is this system-level framework: the episode-and-layer abstraction, the label-free reference library, cross-layer attribution, and a reproducible operational procedure. Within a verified-healthy evaluation window, the tuned configuration achieves an AUROC of 0.97 on the fault classes DTW is mechanically suited to (bias, drift, interaction, and spikes), detecting every injected fault (100% fault-level recall) at a mean delay of 21 min, while constant-value and missing-data faults are shown to require complementary detectors. Operating-point precision and recall are reported across alert budgets (Appendix Table A1); at a 5% false-alarm budget, the system is balanced at about 0.85 precision and recall. These results are based on a single device within a verified-healthy window and on injected faults alongside one real degradation event; generalization to additional devices, modalities, and organically occurring faults remains future work.

## Figures and Tables

**Table 1:** Parameter sensitivity (verified-healthy window, robust-z scorer; CORE AUROC = bias + **drift + interaction)**.

Sweep	Setting	CORE AUROC	+spikes AUROC
Episode length L (min)	30	0.912	0.899
	45	0.944	0.928
	60	0.964	0.945
	90	0.970	0.948
Stride S (min)	10	0.950	n/a
	15	0.939	n/a
	20	0.964	n/a
	30	0.928	n/a
Channel set	HW (4)	0.625	n/a
	All (32)	0.945	n/a
	SW/FW (20)	0.944	n/a
	Non-energy (28)	0.964	n/a

**Table 2:** The confusion matrix at the 95th-percentile alert budget.

		Predicted	
		Positive	Negative
Actual	Positive	81	14
	Negative	14	273

**Table 3:** Per-fault-type results (threshold-independent AUROC).

Fault Type	AUROC	Fault-Level Detection	Mean Delay (min)	DTW Scope
Bias_shift	0.988	100%	10	Appropriate
Interaction	0.989	100%	10	Appropriate
Drift	0.966	100%	35	Appropriate
Spikes	0.934	100%	30	Appropriate
Dropout	0.667	0%	n/a	Out of scope (missingness monitor)
Stuck_at	0.655	0%	n/a	Out of scope (variance/range check)

**Table 4:** Detection summary.

Metric	Value	Source
AUROC (DTW-appropriate, threshold-independent)	0.970	Healthy-window eval
Fault-level detection (DTW-appropriate)	100% (16/16)	Healthy-window eval
Mean detection delay	21.2 min	End-of-episode decision time
Episode-level operating point	See Appendix Table A1	Alert-rate budget
Stuck-at/dropout AUROC	0.66/0.67 (blind spots)	Healthy-window eval

**Table 5:** Suitability of FDD method families.

Method Family	Labeled Faults	Detects Temporal Faults	Cross-Layer Attribution	Interpretable Evidence	Low Deployment Cost
Rule-based	No	No	No	Yes	Yes
Supervised classifiers (SVN, RF, and DNN)	Yes	Only with sequence models	No	Varies by model	No
Isolation Forest/Once-Class SVM	No	No (ordering discarded)	Feature importance only	Score-level only	Yes
Autoencoders	No	Only with temporal architectures	Per-feature error only	No	No
This work	No	Yes	Yes	Yes	Yes

**Figure A1:**
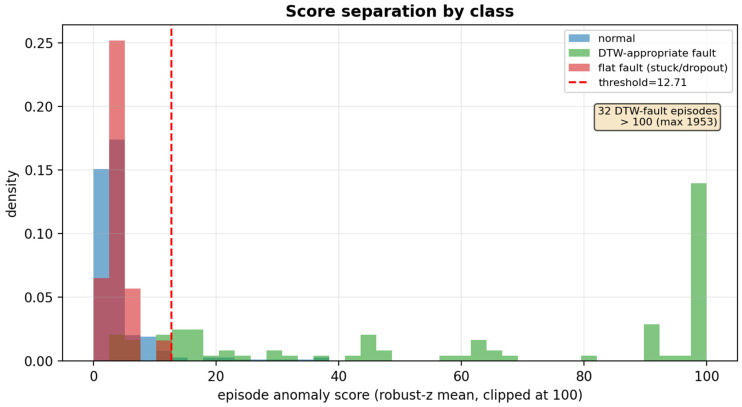
Episode anomaly score distributions by class (robust-z mean over the non-energy channels, clipped at 100 for display), with the 95th-percentile operating point at a threshold of 12.71. The normal episodes and flat faults (stuck-at and dropout) lie predominantly below the threshold, while the DTW-appropriate faults (bias, drift, interaction, and spikes) form a heavy upper tail; 32 DTW-appropriate fault episodes exceed the clipping bound of 100, with a maximum of 1953. The plot shows both the separation of shape-changing faults from normal and the overlap of flat faults with normal, which places them outside the method’s scope.

## Data Availability

Data used in this article can be found at https://db.csail.mit.edu/labdata/labdata.html (accessed on 5 May 2026).

## Appendix A. Operating-Point Trade-Off and Score Separation

**Table A1:** Operating-point trade-off (threshold = percentile of training-normal scores; DTW-appropriate faults versus normal; and verified-healthy window).

Alert Budget (Train-Normal Pct)	Threshold	Precision	Recall	F1	False-Alarm Rate
90th	9.84	0.811	0.905	0.856	0.070
95th	12.71	0.853	0.853	0.853	0.049
97.5th	16.11	0.867	0.758	0.809	0.038
99th	20.27	0.883	0.716	0.791	0.031
